# Laughter is the Best Medicine? A Cross-Sectional Study of Cardiovascular Disease Among Older Japanese Adults

**DOI:** 10.2188/jea.JE20150196

**Published:** 2016-10-05

**Authors:** Kei Hayashi, Ichiro Kawachi, Tetsuya Ohira, Katsunori Kondo, Kokoro Shirai, Naoki Kondo

**Affiliations:** 1 Faculty of Medicine, the University of Tokyo, Tokyo, Japan; 1 東京大学医学部医学科; 2 Department of Social and Behavioral Sciences, Harvard T.H. Chan School of Public Health, Boston, Massachusetts, United States; 2 ハーバード大学公衆衛生大学院; 3 Department of Epidemiology, Fukushima Medical University, Fukushima, Japan; 3 福島県立医科大学 医学部疫学講座; 4 Center for Well-being and Society, Nihon Fukushi University, Chita, Aichi, Japan; 4 日本福祉大学; 5 Center for Preventive Medical Sciences, Chiba University, Chiba, Japan; 5 千葉大学 予防医学センター 社会予防医学研究部門; 6 Department of Human Sciences, School of Law and Letters, University of the Ryukyus, Naha, Japan; 6 琉球大学法文学部人間科学科; 7 Department of Health and Social Behavior/Department of Health Education and Health Sociology, School of Public Health, the University of Tokyo, Tokyo, Japan; 7 東京大学大学院医学系研究科

**Keywords:** laughter, aged, stroke, cardiovascular diseases, Japan, 笑い, 高齢化, 脳卒中, 循環器疾患, 日本

## Abstract

**Background:**

We sought to evaluate the associations between frequency of daily laughter with heart disease and stroke among community-dwelling older Japanese women and men.

**Methods:**

We analyzed cross-sectional data in 20 934 individuals (10 206 men and 10 728 women) aged 65 years or older, who participated in the Japan Gerontological Evaluation Study in 2013. In the mail-in survey, participants provided information on daily frequency of laughter, as well as body mass index, demographic and lifestyle factors, and diagnoses of cardiovascular disease, hyperlipidemia, hypertension, and depression.

**Results:**

Even after adjustment for hyperlipidemia, hypertension, depression, body mass index, and other risk factors, the prevalence of heart diseases among those who never or almost never laughed was 1.21 (95% CI, −1.03–1.41) times higher than those who reported laughing every day. The adjusted prevalence ratio for stroke was 1.60 (95% CI, 1.24–2.06).

**Conclusions:**

Daily frequency of laughter is associated with lower prevalence of cardiovascular diseases. The association could not be explained by confounding factors, such as depressive symptoms.

## Abstract (ja)

**背景：:**

普段の笑いの頻度と心疾患および脳卒中有病の関連について、地域在住の日本の高齢者を対象として調査した。

**方法：:**

日本老年学的評価研究（JAGES）の2013年度の調査の対象者のうち65才以上の20,934人（男性10,206人、女性10,728）のデータを分析した。郵送法の自記式調査により笑いの頻度、BMI、人口統計情報およびライフスタイル関連の要因、循環器疾患、高脂血症、高血圧、抑うつ症状についての情報を収集した。

**結果：:**

高脂血症、高血圧、抑うつ症状、BMI、その他の要因を調整した上でも、ほとんど笑わない群の心疾患の有病割合はほぼ毎日笑う群に比べて1.21倍（95%信頼区間：1.03–1.41） 高かった。 同様に脳卒中の有病割合は1.60倍 （95% 信頼区間：1.24–2.06）高かった。

**結論：:**

普段よく笑う高齢者は循環器疾患（心疾患または脳卒中）が少ないという関連が見られた。この関連は抑うつ症状など要因では説明がつかなかった。

## INTRODUCTION

Laughter is increasingly recognized for its potential health benefits, including ameliorating symptoms of depression,^1^ dementia,^2^ and insomnia.^3^ Several studies have reported beneficial effects of laughter on biomarkers, such as markers of immune function^4^^,^^5^ and HbA_1c_.^6^ Laughter has a role in complementary medicine, such as laughter yoga,^7^ the Smile-Sun technique,^8^ and laughter and exercise programs.^6^

Laughter is also believed to improve vascular function,^9^^,^^10^ but most of these studies have been limited to studying the effect of laughter on intermediate outcomes, such as arterial stiffness and endothelial function. Most of these studies are intervention studies, and the effect of laughter in daily life is unclear. Viachopoulos et al investigated the effects of films-induced laughter on arterial stiffness,^9^ and Miller M et al compared flow-mediated vasodilatation after watching laughter-inducing films and stress-inducing films.^10^

Although rarely, some studies have looked at “hard” health outcomes (eg, actual disease incidence): Adam et al reported that laughter may prevent coronary heart disease (CHD).^11^ They carried out a cross-sectional study using questionnaires to measure responsiveness to situational humor and hostility and found that, even after controlling for CHD risk factors, CHD patients were significantly less likely to experience laughter during daily activities. However, the study sample was only 300 patients, and they did not control for depression, a major confounding factor of laughter. Another study looked at the relationship of laughter with stroke, but it focused on recovery following stroke.^12^

Relatively few epidemiological investigations of the link between laughter and cardiovascular disease have been conducted. In this study, we sought to conduct an analysis of cross-sectional data from a large-scale cohort of community-dwelling Japanese older adults on the association of laughter with cardiovascular disease.

## METHODS

### Study sample

The present study is based on the Japan Gerontological Evaluation Study (JAGES). The JAGES cohort was established in 2010 to investigate factors associated with health and well-being among non-institutionalized individuals aged 65 years and older. The cohort covers 30 municipalities in Japan. We used the 2013 wave of JAGES, in which questionnaires were mailed to 195 290 community-dwelling individuals aged 65 years and older. Of those, 138 294 individuals responded to the survey (response rate, 70.8%). Aside from basic questions, there were five modules of the survey covering different topics. Module A covered nursing care, medical care, and life styles; module B assessed oral hygiene, optimism, and subjective health; module C covered social capital and history of abuse; module D evaluated subjective quality of life, sleep, and cognitive function; and module E assessed physical activity. We used module B, which includes questions about daily frequency of laughter. Respondents to module B comprised 12 174 men and 14 194 women. We excluded 5434 subjects with missing information on subjective health status, frequency of laughing, depression, sex, or age, ultimately including 20 934 participants (10 206 men and 10 728 women).

### Heart diseases and stroke

Our primary objective variables were self-reported history of being diagnosed with heart diseases or stroke. Glymour et al investigated whether self-reported strokes can be used to study stroke incidence and risk factors using the data of Health and Retirement Survey (HRS), which is a self-reported population-based cohort study^13^; the authors found that HRS estimates were closely comparable to those reported in the Cardiovascular Health Study. On the other hand, Bruce et al reported that underreporting and over-reporting of CVD were common among older adults, while the proportions of false-negative self-reports were small.^14^

### Laughter

Daily frequency of laughter was assessed via a standard single-item question^6^: “How often do you laugh out loud?” (almost every day, 1–5 days per week, 1–3 days per month, or never or almost never). We selected “almost every day” as the reference category.

### Covariates

The 2013 survey also inquired about self-reported history of having been diagnosed with hyperlipidemia or hypertension, as well as a range of other personal characteristics, including the presence of depressive symptoms, age, gender, marital status, smoking habit, alcohol consumption, physical activity, and social participation. For evaluation of depressive symptoms, the 15-item Geriatric Depression Scale (GDS-15) was used. The GDS-15 is scored from 1 to 15, with higher scores indicating more depressive symptomatology. Following previous studies, we used 5 as the cutoff score for indicating moderate-to-severe psychological distress.^15^ We inquired about the frequency of participation in different civic associations and social groups. After summing the different forms of social participation for each respondent, we categorized individuals into quartiles, using the bottom quartile as the reference group. For the evaluation of physical activity, we asked about the frequency of any physical activity (eg, running, swimming, cycling, tennis, activities in sports clubs, climbing, walking, dancing, gymnastics, golf, gardening, washing cars, stretching, bowling, and washing clothes) and divided subjects into two groups (less than once per week or once or more per week) and used the latter category as the reference. For the evaluation of smoking habit, we used the standard single-item question: “Do you smoke?” (never or almost never, stopped smoking, or currently smoking). For the evaluation of alcohol consumption, we used the standard single-item question: “Do you drink alcoholic beverages?” (never or almost never, stopped drinking, or currently drinking), and used “never or almost never” as the reference category.

### Statistical analysis

Poisson regression models were used to calculate the prevalence ratios (PRs) and 95% confidence intervals (CIs) for cardiovascular disease according to frequency of laughing. In Model 1, we statistically adjusted for depressive symptoms, age, gender, marital status, smoking habit, alcohol consumption, and physical activity. The variable of depressive symptoms was included in the model because this could confound the association between laughter and cardiovascular disease: depressed people laugh less often, and depression is an independent risk factor for cardiovascular disease.^1^ We also controlled for hypertension and hyperlipidemia in the models when heart disease or stroke was the outcome of interest. In these models, hypertension and hyperlipidemia are not necessarily confounding variables but may be related to cardiovascular disease. For all explanatory variables, we set categories that were expected to confer the least health risk as referent categories, based on existing evidence and our hypotheses.

In Model 2, we added social participation as a potential confounder, because social participation could increase the frequency of opportunities for laughter and is an independent inversely associated factor for cardiovascular disease.^16^ For instance, if the risk ratio for cardiovascular disease becomes attenuated towards the null, we would conclude that social participation is more strongly related to cardiovascular disease than laughter. All statistical analyses were conducted using R version 3.1.0 (R Foundation for Statistical Computing, Vienna, Austria).

### Ethical issues

Our study protocol and informed consent procedure were approved by the Ethics Committee on the Research of Human Subjects at Nihon Fukushi University.

## RESULTS

Baseline characteristics are shown in Table 1. The prevalence of self-reported heart disease was higher (2238 cases; 10.7%) than that of stroke (676 cases; 3.2%). The prevalence of hypertension was much higher than that of the other three diseases (8998 cases; 42.9%). People who reported having been diagnosed with stroke or hypertension had lower frequency of laughter; however, this was not true for those who reported being diagnosed with hyperlipidemia. People tend to laugh more often if they participated in social activities more frequently, smoked less, drank more, exercised more frequently, and had higher BMI.

**Table 1.** Frequency of laughing in 4 weeks by participants’ characteristics

	*n*	Never or almost never (%)	1–3 days per month (%)	1–5 days per week (%)	Almost everyday (%)
**Cardiovascular diseases (%)**					
Heart diseases	2238	242 (15.2%)	320 (12.9%)	863 (11.0%)	813 (9.0%)
Stroke	676	102 (6.4%)	104 (4.2%)	244 (3.1%)	226 (2.5%)
**Risk factor diseases (%)**					
Hyperlipidemia	2534	158 (9.9%)	307 (12.4%)	964 (12.3%)	1105 (12.2%)
Hypertension	8998	700 (44.0%)	1067 (43.2%)	3381 (43.1%)	3850 (42.6%)
**Depression (%)**					
GDS score ≥5	3232	715 (44.9%)	635 (25.7%)	1198 (15.3%)	684 (7.6%)
GDS score <5	17 702	877 (55.1%)	1837 (74.3%)	6639 (84.7%)	8349 (92.4%)
**Age, years (%)**					
65–69	6305	397 (24.9%)	706 (28.6%)	2376 (30.3%)	2826 (31.3%)
70–74	6408	423 (26.6%)	693 (28.0%)	2342 (29.9%)	2950 (32.7%)
75–79	4498	359 (22.6%)	563 (22.8%)	1675 (21.4%)	1901 (21.0%)
≥80	3723	413 (25.9%)	510 (20.6%)	1444 (18.4%)	1356 (15.0%)
**Body mass index**					
1st quintile	4010	365	506	1547	1592
2nd quintile	4017	277	470	1563	1707
3rd quintile	4116	303	458	1582	1773
4th quintile	3917	255	467	1399	1796
5th quintile	3989	293	453	1433	1810
Missing data	885	99	118	313	355
**Alcohol consumption (%)**					
Never or almost never	12 118	860 (54.0%)	1265 (51.2%)	4498 (57.4%)	5495 (60.8%)
Stopped drinking	1045	137 (8.6%)	175 (7.1%)	380 (4.8%)	353 (3.9%)
Currently Drinking	7538	584 (36.7%)	983 (39.8%)	2868 (36.6%)	3103 (34.4%)
Missing data	233	11 (0.7%)	49 (2.0%)	91 (1.2%)	82 (0.9%)
**Smoking habit (%)**					
Never or almost never	15 025	973 (61.1%)	1570 (63.5%)	5609 (71.6%)	6873 (76.1%)
Stopped smoking	3410	336 (21.1%)	513 (20.8%)	1312 (16.7%)	1249 (13.8%)
Currently smoking	2244	267 (16.8%)	342 (13.8%)	831 (10.6%)	804 (8.9%)
Missing data	255	16 (1.0%)	47 (1.9%)	85 (1.1%)	107 (1.2%)
**Physical activity (%)**					
Less than once per week	3173	492 (30.9%)	526 (21.3%)	1078 (13.8%)	1077 (11.9%)
Once or more per week	17 761	1100 (69.1%)	1946 (78.7%)	6759 (86.2%)	7956 (88.1%)
Missing data	2888	234 (14.7%)	301 (12.2%)	1073 (13.7%)	1280 (14.2%)
**Frequency of social participation per year**					
1st quartile	4623	663	629	1639	1692
2nd quartile	3699	294	501	1405	1499
3rd quartile	3764	207	479	1413	1665
4th quartile	3882	122	297	1514	1949
Missing data	4966	306	566	1866	2228

GDS, Geriatric Depression Scale.

The prevalence of cardiovascular disease according to daily frequency of laughter is shown in Table 2. For cardiovascular diseases (heart disease and stroke), a clear dose-response gradient was observed among both men and women between the daily frequency of laughter and the prevalence of disease. The results of Poisson regression models linking laughter and cardiovascular disease outcomes are shown in Table 3. In the crude model, we found an association between daily frequency of laughter and both heart disease and stroke. Compared to the PR of those who laughed almost every day, the PR of people who never or almost never laughed was 1.69 (95% CI, 1.46–1.95) for heart diseases and 2.56 (95% CI, 2.03–3.24) for stroke. Although these PRs were attenuated by the successive addition of covariates (in models 1 and 2), even in the fully adjusted model (model 2), we found associations between daily frequency of laughter and the two cardiovascular diseases, with adjusted PRs of 1.21 (95% CI, 1.03–1.41) for heart disease and 1.60 (95% CI, 1.24–2.06) for stroke. We also found that depression was associated with increased risks of both stroke (PR 1.37; 95% CI, 1.23–1.52) and heart disease (PR 1.39; 95% CI, 1.15–1.68). Social participation had an inverse association with these cardiovascular diseases, and smoking was somehow associated with decreased risk of heart disease (PR 0.72; 95% CI, 0.61–0.85).

**Table 2.** Frequency of laughing in 4 weeks by participants’ status of cardiovascular diseases and risk factor diseases

	*n*	Never or almost never (%)	1–3 days per month (%)	1–5 days per week (%)	Almost everyday (%)
**Men**					
Cardiovascular diseases					
Heart diseases	1382	16.4	15.0	13.2	12.5
Stroke	460	7.6	5.5	4.0	3.8
Cardiovascular risk factors					
Hyperlipidemia	974	8.5	10.5	9.6	9.4
Hypertension	4361	42.9	43.0	42.6	42.7
**Women**					
Cardiovascular diseases					
Heart diseases	856	13.0	9.8	8.9	6.4
Stroke	216	4.2	2.2	2.3	1.6
Cardiovascular risk factors					
Hyperlipidemia	1560	12.5	15.4	14.9	14.3
Hypertension	4637	46.0	43.4	43.6	42.6

**Table 3.** Prevalence ratio and confidence intervals for heart diseases and stroke

Variable	Crude Models		Model 1		Model 2	
		
	Heart diseases	Stroke	Heart diseases	Stroke	Heart disease	Stroke
	PR (95% CI)	PR (95% CI)	PR (95% CI)	PR (95% CI)	PR (95% CI)	PR (95% CI)
**Risk factor diseases**						
Hypertension	0.98 (0.87–1.12)	0.93 (0.74–1.17)	1.10 (0.97–1.25)	1.07 (0.85–1.35)	1.10 (0.97–1.25)	1.08 (0.86–1.37)
Hyperlipidemia	1.50 (1.38–1.64)	1.95 (1.67–2.28)	1.38 (1.27–1.51)	1.84 (1.57–2.15)	1.38 (1.27–1.51)	1.83 (1.56–2.14)
**Frequency of laughing per month**						
Never or almost never	1.69 (1.46–1.95)	2.56 (2.03–3.24)	1.21 (1.04–1.41)	1.67 (1.30–2.15)	1.21 (1.03–1.41)	1.60 (1.24–2.06)
1–3 days per month	1.44 (1.26–1.64)	1.68 (1.33–2.12)	1.18 (1.03–1.35)	1.28 (1.01–1.63)	1.18 (1.03–1.35)	1.27 (1.00–1.61)
1–5 days per week	1.22 (1.11–1.35)	1.24 (1.04–1.49)	1.13 (1.03–1.25)	1.12 (0.93–1.34)	1.13 (1.03–1.25)	1.12 (0.93–1.34)
Almost everyday	Ref	Ref	Ref	Ref	Ref	Ref
**Depression**						
GDS score ≥5	1.57 (1.42–1.74)	1.84 (1.55–2.19)	1.37 (1.23–1.52)	1.45 (1.20–1.75)	1.37 (1.23–1.52)	1.39 (1.15–1.68)
GDS score <5	Ref	Ref	Ref	Ref	Ref	Ref
**Age, years**						
65–69	Ref	Ref	Ref	Ref	Ref	Ref
70–74	1.60 (1.42–1.82)	1.35 (1.09–1.68)	1.56 (1.38–1.77)	1.31 (1.05–1.63)	1.56 (1.38–1.77)	1.31 (1.05–1.63)
75–79	2.04 (1.80–2.32)	1.76 (1.41–2.20)	1.89 (1.66–2.15)	1.57 (1.25–1.96)	1.90 (1.66–2.16)	1.55 (1.23–1.94)
≥80	2.53 (2.23–2.87)	2.03 (1.62–2.54)	2.29 (2.00–2.61)	1.74 (1.37–2.19)	2.29 (2.01–2.61)	1.68 (1.33–2.12)
**Gender**						
Men	1.70 (1.56–1.85)	2.24 (1.90–2.63)	1.87 (1.69–2.07)	2.06 (1.69–2.51)	1.87 (1.68–2.07)	2.08 (1.71–2.54)
Women	Ref	Ref	Ref	Ref	Ref	Ref
**Body mass index**						
1st quintile	0.92 (0.80–1.06)	0.80 (0.63–1.02)	0.99 (0.86–1.14)	0.92 (0.72–1.18)	0.99 (0.86–1.14)	0.91 (0.71–1.16)
2nd quintile	0.93 (0.81–1.07)	0.84 (0.66–1.07)	0.97 (0.85–1.12)	0.93 (0.73–1.18)	0.98 (0.85–1.12)	0.93 (0.73–1.18)
3rd quintile	Ref	Ref	Ref	Ref	Ref	Ref
4th quintile	1.08 (0.95–1.24)	0.89 (0.70–1.13)	1.08 (0.94–1.23)	0.87 (0.69–1.11)	1.08 (0.94–1.23)	0.87 (0.69–1.11)
5th quintile	1.29 (1.13–1.46)	1.03 (0.82–1.30)	1.25 (1.10–1.42)	0.97 (0.77–1.22)	1.25 (1.10–1.42)	0.96 (0.76–1.21)
Missing data	1.08 (0.87–1.35)	0.83 (0.55–1.26)	0.94 (0.75–1.18)	0.73 (0.48–1.11)	0.94 (0.76–1.18)	0.72 (0.47–1.10)
**Alcohol consumption**						
Never or almost never	Ref	Ref	Ref	Ref	Ref	Ref
Stopped drinking	1.63 (1.40–1.91)	2.73 (2.13–3.50)	1.06 (0.89–1.26)	1.55 (1.18–2.05)	1.06 (0.89–1.25)	1.54 (1.17–2.04)
Currently drinking	0.95 (0.86–1.03)	1.29 (1.10–1.52)	0.75 (0.67–0.83)	0.93 (0.77–1.12)	0.75 (0.67–0.83)	0.95 (0.79–1.14)
Missing data	0.73 (0.46–1.16)	1.43 (0.74–2.78)	0.61 (0.32–1.14)	1.92 (0.76–4.88)	0.61 (0.32–1.15)	1.93 (0.76–4.90)
**Smoking habit**						
Never or almost never	Ref	Ref	Ref	Ref	Ref	Ref
Stopped smoking	1.43 (1.30–1.59)	1.89 (1.59–2.25)	1.10 (0.98–1.24)	1.17 (0.96–1.44)	1.10 (0.98–1.24)	1.17 (0.96–1.43)
Currently smoking	0.80 (0.69–0.93)	1.07 (0.82–1.38)	0.72 (0.61–0.85)	0.80 (0.61–1.06)	0.72 (0.61–0.85)	0.79 (0.60–1.04)
Missing data	0.88 (0.59–1.33)	0.98 (0.46–2.07)	1.05 (0.60–1.83)	0.53 (0.18–1.51)	1.05 (0.60–1.84)	0.52 (0.18–1.50)
**Physical activity**						
Less than once per week	1.44 (1.29–1.59)	1.76 (1.47–2.12)	1.15 (1.03–1.28)	1.32 (1.09–1.60)	1.14 (1.02–1.27)	1.25 (1.03–1.52)
Once or more per week	Ref	Ref	Ref	Ref	Ref	Ref
Missing data	1.08 (0.96–1.22)	1.30 (1.05–1.61)	0.96 (0.85–1.09)	1.22 (0.98–1.52)	0.97 (0.86–1.11)	1.17 (0.94–1.47)
**Frequency of social participation per year**						
1st quartile	1.31 (1.15–1.49)	1.95 (1.52–2.50)			1.02 (0.89–1.17)	1.43 (1.11–1.86)
2nd quartile	1.12 (0.97–1.29)	1.45 (1.10–1.91)			0.96 (0.83–1.10)	1.18 (0.89–1.56)
3rd quartile	1.10 (0.96–1.27)	1.08 (0.81–1.44)			1.01 (0.88–1.17)	0.95 (0.71–1.27)
4th quartile	Ref	Ref			Ref	Ref
Missing data	1.07 (0.94–1.23)	1.44 (1.11–1.86)			0.95 (0.83–1.09)	1.28 (0.98–1.66)

CI, confidence interval; GDS, Geriatric Depression Scale; PR, prevalence ratio.

In model 1, we controlled for risk factors of diseases, laughter, depression, age, gender, body mass index, drinking habit, smoking habit, and physical activity.

In model 2, social participation was added to the variables.

Gender-stratified analyses showed almost the same results (eTable 1, eTable 2, eTable 3, eTable 4, and eTable 5). Men had almost twice the prevalence of cardiovascular disease as women, and women laughed more frequently than men (eTable 1). Men also smoked much more, drank more, and were twice as likely to be sedentary (exercising less than once per week). However, men had about the same level of depressive symptoms as women. Men and women also participated in social activities to roughly the same extent.

## DISCUSSION

In this cross-sectional study, we found inverse associations between daily frequency of laughter and a self-reported history of having been diagnosed with heart disease or stroke. Risk estimates were attenuated in models adjusting for potential confounders, such as depressive symptoms and extent of social participation. Nonetheless, even in the fully-adjusted models, individuals who reported almost never laughing had a prevalence of heart disease that was 1.21 (95% CI, 1.03–1.41) times higher than those who laughed almost every day. Similarly, the prevalence of stroke was 1.60 (95% CI, 1.24–2.06) times higher among people who reported rarely laughing.

Various mechanisms may account for the association between laughter and heart disease or stroke. First, laughter is known to buffer the effects of psychological stress,^17^ which is proposed as a major risk factor for cardiovascular disease.^18^^–^^22^ There is evidence that laughter can reduce stress. Kim et al reported that laughter therapy significantly decreased the severity of depression, anxiety, and perceived stress in an experimental group who received laughter therapy compared to those in the control group.^23^ Bennett et al reported that laughter reduced stress levels and improved natural killer cell activity compared with those assigned to a control condition.^24^ Second, laughter improves vascular endothelial function,^9^^,^^10^ improving arterial compliance^25^ and attenuating neuroendocrine hormones involved in the down-regulation of vasodilatation.^10^ Although evidence is not sufficient at present, laughter may function as a form of exercise or physical activity, which is an important preventive factor for heart disease^26^ and stroke.^27^

Caution is warranted in interpreting our findings. First, our study is cross-sectional, so we cannot rule out “reverse causality”, in which people diagnosed with serious illnesses (such as stroke and heart disease) may experience fewer occasions in daily life to feel cheerful. Reverse causation is also applicable in the case of stroke, which may be associated with complications, such as facial paralysis, which may impair people’s ability to laugh.^28^^,^^29^ A definitive answer to the question of temporal sequence must await prospective follow-up of the JAGES cohort to observe incident cardiovascular events. Second, laughter may itself be a marker or proxy of physically and/or mentally positive lifestyles. People who have a more positive outlook on life may be more motivated to engage in healthy behaviors, such as exercise, healthy diet, and moderation in alcohol consumption. Although we controlled for many of these behaviors, the possibility of residual confounding cannot be ruled out. Third, our objective and explanatory variables were self-reported. Although some studies in the United States reported that self-reported information is valid enough to be used in epidemiologic studies, their findings might not be applicable to the Japanese data we used. Validation studies using Japanese data are warranted. Nonetheless, we have some confidence in our results because most of the established risk factors for cardiovascular disease indicated associations in the expected direction (eg, higher BMI, sedentarism, and depression).^28^^–^^30^ The major exception is smoking, which was associated with decreased prevalence of cardiovascular disease in our analysis.^31^ We think this is mainly due to some deviation in our data: in the JAGES 2013 cross-sectional data, 8787 of 131 920 participants did not report any specific disease but also did not select “do not have any disease”, which may have affected results. Also we excluded 5434 participants with missing data on laughter or depression out of 26 368 total participants. Another possible reason is that some participants may have chosen “smoking” or “never or almost never” arbitrarily, since they were not sure in which category they belonged. Reverse causation may also explain this unexpected finding, since some participants who were diagnosed with heart disease would have stopped smoking. There are many recently developed devices to measure laughter^30^^,^^31^; although self-reported laughter may not be as reliable as these measurement methods, the directions of the associations with cardiovascular disease are consistent with those of previous studies. Lastly, we did not consider different types of laughter. There are many types of laughing; for example, smiling is an indication of fondness and appeasement, while laughter expresses playfulness.^31^ Duchenne laughter arises from positive emotions, whereas non-Duchenne laughter is not based on humor or positive emotions.^32^ Further studies are needed to examine the differential impacts according to types of laughter, although this would be difficult to do in a large-scale epidemiological study (where external observation is not possible).

In conclusion, if laughter has inverse associations with cardiovascular disease onset, it would be useful to develop interventions to promote laughter in people’s lives (eg, laughter therapy). Population-based interventions, such as increasing opportunities for social interactions in the community, are also required. Although our study could not clearly show any preventive effect of laughter on cardiovascular diseases due to its cross-sectional nature, the present findings are consistent with such an effect, since those who reported having been diagnosed with stroke or heart disease were found not to laugh as often as those who did not have a history of stroke or heart disease. The mechanisms linking laughter and cardiovascular diseases warrant further study. For instance, a longitudinal study with devices to measure daily laughter may be able to evaluate the preventive effect of laughter on cardiovascular diseases.

## ONLINE ONLY MATERIALS

eTable 1. Characteristics of the subjects by gender.

eTable 2. Frequency of laughing in 4 weeks by participants’ characteristics in men.

eTable 3. Frequency of laughing in 4 weeks by participants’ characteristics in women.

eTable 4. Prevalence ratio and confidence intervals for heart diseases and stroke in men.

eTable 5. Prevalence ratio and confidence intervals for heart diseases and stroke in women.

Abstract in Japanese.
