# Supplementary material for: Laughter is the Best Medicine? A Cross-Sectional Study of Cardiovascular Disease Among Older Japanese Adults
Source: J Epidemiol. 2016 Oct 5;26(10):546–52. doi: 10.2188/jea.JE20150196 (PMC5037252; doi:10.2188/jea.JE20150196)
Supplement: eTable 1. [file je-26-546-s001.pdf]

eTable 1. Characteristics of the subjects by gender

	Men (n=10,206)	Women (n=10,728)
**Cardiovascular diseases (%)**		
Heart diseases	1,382 (13.5%)	856 (8.0%)
Stroke	460 (4.5%)	216 (2.0%)
**Risk factor diseases (%)**		
Hyperlipidemia	974 (9.5%)	1,560 (14.5%)
Hypertension	4,361 (42.7%)	4,637 (43.2%)
**Frequency of laughing in 4 weeks (%)**		
Never or almost never	1,022 (10.0%)	570 (5.3%)
1-3 days per month	1,509 (14.8%)	963 (9.0%)
1-5 days per week	3,846 (37.7%)	3,991 (37.2%)
Almost everyday	3,829 (37.5%)	5,204 (48.5%)
**Depression (%)**		
GDS score ≥5	1,555 (15.2%)	1,677 (15.6%)
GDS score <5	8,651 (84.8%)	9,051 (84.4%)
**Age, years (%; mean [SD])**		
65-69	3,162 (31.0%)	3,143 (29.3%)
70-74	3,084 (30.2%)	3,324 (31.0%)
75-79	2,207 (21.6%)	2,291 (21.4%)
≥80	1,753 (17.2%)	1,970 (18.4%)
Mean (SD)	73.42 (6.01)	73.69 (6.15)
**Body mass index (%; mean [SD])**[a]		
Continuous: Mean (SD)	23.11 (2.90)	22.64 (3.31)
1st quintile	1,998 (19.6%)	2,045 (19.1%)
2nd quintile	1,944 (19.0%)	2,047 (19.1%)
3rd quintile	1,972 (19.3%)	2,051 (19.1%)
4th quintile	2,031 (19.9%)	2,018 (18.8%)
5th quintile	1,903 (18.6%)	2,040 (19.0%)
Missing data	358 (3.5%)	527 (4.9%)
**Alcohol consumption (%)**		
Never or almost never	3,437 (33.7%)	8,681 (80.9%)
Stopped drinking	848 (8.3%)	197 (1.8%)
Drinking	5,820 (57.0%)	1,718 (16.0%)
Missing data	101 (1.0%)	132 (1.2%)
**Smoking habit (%)**		
Never or almost never	5,067 (49.6%)	9,958 (92.8%)

Stopped smoking	3,159 (31.0%)	251 (2.3%)
Currently smoking	1,871 (18.3%)	373 (3.5%)
Missing data	109 (1.1%)	146 (1.4%)
**Physical activity (%)**		
Less than once per week	2,054 (20.1%)	1,119 (10.4%)
Once or more per week	7,038 (69.0%)	7,835 (73.0%)
Missing data	1,114 (10.9%)	1,774 (16.5%)
**Frequency of social participation per year (%; median [25%, 75%])[b]**		
Median [25%, 75%]	24 [0, 102]	24 [0, 132]
1st quartile	2,327 (22.8%)	2,296 (21.4%)
2nd quartile	2,087 (20.4%)	1,612 (15.0%)
3rd quartile	1,767 (17.3%)	1,907 (17.8%)
4th quartile	2,040 (20.0%)	1,932 (18.0%)
Missing data	1,985 (19.4%)	2,981 (27.8%)

CI, confidence interval; GDS, Geriatric Depression Scale; SD, standard deviation.

[a]1st quintile (Men: 13.52 - 20.76, Women: 6.67 - 19.92); 2nd quintile (Men: 20.76 - 22.32, Women:19.92- 21.49); 3rd quintile (Men: 22.32 - 23.63., Women: 21.49 - 23.12); 4th quintile (Men: 23.63 - 25.39, Women: 23.12 - 25.20); 5th quintile (Men: 25.39 - 36.21, Women: 25.20 - 41.42)

[b]1st quartile (Men: 0 times, Women: 0 times); 2nd quartile (Men: 1-24 times, Women: 1-24 times); 3rd quartile (Men: 25-102 times, Women: 25-132 times); 4th quartile (Men: 103-2640 times, Women: 133-2004 times or more)
