# Supplementary material for: Laughter is the Best Medicine? A Cross-Sectional Study of Cardiovascular Disease Among Older Japanese Adults
Source: J Epidemiol. 2016 Oct 5;26(10):546–52. doi: 10.2188/jea.JE20150196 (PMC5037252; doi:10.2188/jea.JE20150196)
Supplement: eTable 2. [file je-26-546-s002.pdf]

eTable 2. Frequency of laughing in 4 weeks by participants' characteristics in men

	n	Never or almost never (% yes)	1-3 days per month (% yes)	1-5 days per week (% yes)	Almost everyday (% yes)
**Cardiovascular diseases (%)**					
Heart diseases	1,382	168 (16.4%)	226 (15.0%)	508 (13.2%)	480 (12.5%)
Stroke	460	78 (7.6%)	83 (5.5%)	154 (4.0%)	145 (3.8%)
**Risk factor diseases (%)**					
Hyperlipidemia	974	87 (8.5%)	159 (10.5%)	369 (9.6%)	359 (9.4%)
Hypertension	4,361	438 (42.9%)	649 (43.0%)	1,640 (42.6%)	1,634 (42.7%)
**Depression (%)**					
GDS score ≥5	1,555	429 (42.0%)	334 (22.1%)	505 (13.1%)	287 (7.5%)
GDS score <5	8,651	593 (58.0%)	1,175 (77.9%)	3,341 (86.9%)	3,542 (92.5%)
**Age, years (%)**					
6-69	3,162	272 (26.6%)	459 (30.4%)	1,220 (31.7%)	1,211 (31.6%)
70-74	3,084	269 (26.3%)	405 (26.8%)	1,154 (30.0%)	1,256 (32.8%)
75-79	2,207	240 (23.5%)	344 (22.8%)	804 (20.9%)	819 (21.4%)
≥80	1,753	241 (23.6%)	301 (19.9%)	668 (17.4%)	543 (14.2%)
**Body mass index**					
1st quintile	1,998	254	327	770	647
2nd quintile	1,944	197	311	745	691
3rd quintile	1,972	172	265	781	754
4th quintile	2,031	169	284	729	849
5th quintile	1,903	176	263	700	764
Missing data	358	54	59	121	124
**Alcohol consumption (%)**					
Never or almost never	3,437	375 (36.7%)	498 (33.0%)	1,272 (33.1%)	1,292 (33.7%)
Stopped drinking	848	121 (11.8%)	153 (10.1%)	305 (7.9%)	269 (7.0%)
Drinking	5,820	518 (50.7%)	831 (55.1%)	2,230 (58.0%)	2,241 (58.5%)
Missing data	101	8 (0.8%)	27 (1.8%)	39 (1.0%)	27 (0.7%)
**Smoking habit (%)**					
Never or almost never	5,067	473 (46.3%)	687 (45.5%)	1,907	2,000

				(49.6%)	(52.2%)
Stopped smoking	3,159	318 (31.1%)	493 (32.7%)	1,204 (31.3%)	1,144 (29.9%)
Currently smoking	1,871	222 (21.7%)	305 (20.2%)	698 (18.1%)	646 (16.9%)
Missing data	109	9 (0.9%)	24 (1.6%)	37 (1.0%)	39 (1.0%)
**Physical activity (%)**					
Less than once per week	2,054	339 (33.2%)	378 (25.0%)	681 (17.7%)	656 (17.1%)
Once or more per week	8,152	683 (66.8%)	1131 (75.0%)	3165 (82.3%)	3173 (82.9%)
Missing data	1,114	119 (11.6%)	141 (9.3%)	419 (10.9%)	435 (11.4%)
**Frequency of social participation per year**					
1st quartile	2,327	417	355	817	738
2nd quartile	2,087	216	343	771	757
3rd quartile	1,767	130	283	680	674
4th quartile	2,040	96	223	829	892
Missing data	1,985	163	305	749	768

GDS, Geriatric Depression Scale.
