# Supplementary material for: Laughter is the Best Medicine? A Cross-Sectional Study of Cardiovascular Disease Among Older Japanese Adults
Source: J Epidemiol. 2016 Oct 5;26(10):546–52. doi: 10.2188/jea.JE20150196 (PMC5037252; doi:10.2188/jea.JE20150196)
Supplement: eTable 3. [file je-26-546-s003.pdf]

**eTable 3**. Frequency of laughing in 4 weeks by participants' characteristics in women

| | N | Never or almost never (% yes) | 1-3 days per month (% yes) | 1-5 days per week (% yes) | Almost everyday (% yes) |
|---|---|---|---|---|---|
| **Cardiovascular diseases (%)** | | | | | |
| Heart diseases | 856 | 74 (13.0%) | 94 (9.8%) | 355 (8.9%) | 333 (6.4%) |
| Stroke | 216 | 24 (4.2%) | 21 (2.2%) | 90 (2.3%) | 81 (1.6%) |
| **Risk factor diseases (%)** | | | | | |
| Hyperlipidemia | 1,560 | 71 (12.5%) | 148 (15.4%) | 595 (14.9%) | 746 (14.3%) |
| Hypertension | 4,637 | 262 (46.0%) | 418 (43.4%) | 1,741 (43.6%) | 2,216 (42.6%) |
| **Depression (%)** | | | | | |
| GDS score ≥5 | 1,677 | 286 (50.2%) | 301 (31.3%) | 693 (17.4%) | 397 (7.6%) |
| GDS score <5 | 9,051 | 284 (49.8%) | 662 (68.7%) | 3,298 (82.6%) | 4,807 (92.4%) |
| **Age, years (%)** | | | | | |
| 65-69 | 3,143 | 125 (21.9%) | 247 (25.6%) | 1,156 (29.0%) | 1,615 (31.0%) |
| 70-74 | 3,324 | 154 (27.0%) | 288 (29.9%) | 1,188 (29.8%) | 1,694 (32.6%) |
| 75-79 | 2,291 | 119 (20.9%) | 219 (22.7%) | 871 (21.8%) | 1,082 (20.8%) |
| ≥80 | 1,970 | 172 (30.2%) | 209 (21.7%) | 776 (19.4%) | 813 (15.6%) |
| **Body mass index** | | | | | |
| 1st quintile | 2,045 | 136 | 209 | 780 | 920 |
| 2nd quintile | 2,047 | 92 | 163 | 816 | 976 |
| 3rd quintile | 2,051 | 103 | 168 | 779 | 1,001 |
| 4th quintile | 2,018 | 89 | 186 | 698 | 1,045 |
| 5th quintile | 2,040 | 105 | 178 | 726 | 1,031 |
| Missing data | 527 | 45 | 59 | 192 | 231 |
| **Alcohol consumption (%)** | | | | | |
| Never or almost never | 8,681 | 485 (85.1%) | 767 (79.6%) | 3226 (80.8%) | 4203 (80.8%) |
| Stopped drinking | 197 | 16 (2.8%) | 22 (2.3%) | 75 (1.9%) | 84 (1.6%) |
| Drinking | 1,718 | 66 (11.6%) | 152 (15.8%) | 638 (16.0%) | 862 (16.6%) |
| Missing data | 132 | 3 (0.5%) | 22 (2.3%) | 52 (1.3%) | 55 (1.1%) |
| **Smoking habit (%)** | | | | | |

| | | | | | |
|---|---|---|---|---|---|
| Never or almost never | 9,958 | 500 (87.7%) | 883 (91.7%) | 3,702 (92.8%) | 4,873 (93.6%) |
| Stopped smoking | 251 | 18 (3.2%) | 20 (2.1%) | 108 (2.7%) | 105 (2.0%) |
| Currently smoking | 373 | 45 (7.9%) | 37 (3.8%) | 133 (3.3%) | 158 (3.0%) |
| Missing data | 146 | 7 (1.2%) | 23 (2.4%) | 48 (1.2%) | 68 (1.3%) |
| **Physical activity (%)** | | | | | |
| Less than once per week | 1,119 | 153 (26.8%) | 148 (15.4%) | 397 (9.9%) | 421 (8.1%) |
| Once or more per week | 9,609 | 417 (73.2%) | 815 (84.6%) | 3,594 (90.1%) | 4,783 (91.9%) |
| Missing data | 1,774 | 115 (20.2%) | 160 (16.6%) | 654 (16.4%) | 845 (16.2%) |
| **Frequency of social participation per year** | | | | | |
| 1st quartile | 2,296 | 246 | 274 | 822 | 954 |
| 2nd quartile | 1,612 | 78 | 158 | 634 | 742 |
| 3rd quartile | 1,907 | 60 | 170 | 687 | 990 |
| 4th quartile | 1,932 | 43 | 100 | 731 | 1,058 |
| Missing data | 2,981 | 143 | 261 | 1,117 | 1,460 |

GDS, Geriatric Depression Scale.
