# Supplementary material for: Laughter is the Best Medicine? A Cross-Sectional Study of Cardiovascular Disease Among Older Japanese Adults
Source: J Epidemiol. 2016 Oct 5;26(10):546–52. doi: 10.2188/jea.JE20150196 (PMC5037252; doi:10.2188/jea.JE20150196)
Supplement: eTable 5. [file je-26-546-s005.pdf]

eTable 5. Prevalence ratio and confidence intervals for heart diseases and stroke in women

	Crude Models		Model 1		Model 2	
	Heart diseases	Stroke	Heart diseases	Stroke	Heart disease	Stroke
Variable	PR (95%CI)	PR (95%CI)	PR (95%CI)	PR (95%CI)	PR (95%CI)	PR (95%CI)
**Cardiovascular diseases**						
Hypertension	0.91 (0.75-1.10)	0.78 (0.52-1.18)	1.00 (0.82-1.22)	0.83 (0.55-1.26)	1.00 (0.82-1.22)	0.85 (0.56-1.28)
Hyperlipidemia	1.68 (1.47-1.93)	1.54 (1.17-2.01)	1.48 (1.29-1.70)	1.34 (1.02-1.77)	1.48 (1.29-1.70)	1.34 (1.02-1.77)
**Frequency of laughing per month**						
Never or almost never	2.03 (1.58-2.61)	2.71 (1.72-4.27)	1.42 (1.08-1.85)	2.00 (1.23-3.27)	1.41 (1.08-1.85)	1.95 (1.19-3.18)
1-3 days per month	1.53 (1.21-1.92)	1.40 (0.87-2.26)	1.26 (1.00-1.59)	1.22 (0.74-1.98)	1.26 (1.00-1.59)	1.19 (0.73-1.95)
1-5 days per week	1.39 (1.20-1.61)	1.45 (1.07-1.96)	1.28 (1.10-1.48)	1.34 (0.99-1.82)	1.28 (1.10-1.49)	1.33 (0.98-1.81)
Almost everyday	Ref	Ref	Ref	Ref	Ref	Ref
**Depression**						
GDS score ≥5	1.77 (1.51-2.06)	1.71 (1.25-2.34)	1.42 (1.20-1.68)	1.26 (0.90-1.77)	1.42 (1.20-1.68)	1.25 (0.89-1.76)
GDS score <5	Ref	Ref	Ref	Ref	Ref	Ref
**Age, years**						
65-69	Ref	Ref	Ref	Ref	Ref	Ref
70-74	1.92 (1.55-2.39)	1.12 (0.75-1.67)	1.84 (1.48-2.29)	1.12 (0.75-1.67)	1.84 (1.48-2.29)	1.09 (0.73-1.63)
75-79	2.45 (1.96-3.06)	1.75 (1.18-2.59)	2.21 (1.76-2.77)	1.68 (1.12-2.52)	2.22 (1.77-2.78)	1.63 (1.08-2.44)
≥80	3.50 (2.83-4.34)	2.32 (1.58-3.41)	2.94 (2.35-3.67)	2.07 (1.39-3.10)	2.94 (2.35-3.68)	1.96 (1.31-2.94)
**Body mass index**						
1st quintile	1.11 (0.89-1.38)	0.70 (0.45-1.08)	1.10 (0.88-1.38)	0.67 (0.43-1.04)	1.10 (0.88-1.38)	0.66 (0.43-1.03)
2nd quintile	0.97 (0.77-1.22)	0.65 (0.42-1.02)	0.99 (0.79-1.24)	0.66 (0.42-1.03)	0.99 (0.79-1.24)	0.66 (0.42-1.03)
3rd quintile	Ref	Ref	Ref	Ref	Ref	Ref
4th quintile	1.12 (0.90-1.39)	0.79 (0.52-1.20)	1.12 (0.90-1.40)	0.81 (0.53-1.23)	1.12 (0.90-1.40)	0.81 (0.53-1.24)
5th quintile	1.21 (0.98-1.50)	1.05 (0.71-1.55)	1.12 (0.90-1.40)	0.99 (0.66-1.46)	1.13 (0.91-1.40)	0.98 (0.66-1.46)

Missing data	1.13 (0.81-1.59)	0.95 (0.51-1.79)	0.89 (0.64-1.25)	0.75 (0.40-1.42)	0.89 (0.64-1.25)	0.74 (0.39-1.40)
**Alcohol consumption**						
Never or almost never	Ref	Ref	Ref	Ref	Ref	Ref
Stopped drinking	1.53 (1.03-2.28)	1.50 (0.67-3.39)	1.63 (1.08-2.46)	1.01 (0.43-2.37)	1.61 (1.07-2.44)	0.99 (0.42-2.35)
Drinking	0.69 (0.56-0.85)	0.86 (0.58-1.27)	0.80 (0.64-0.99)	0.89 (0.59-1.32)	0.80 (0.64-0.99)	0.90 (0.60-1.35)
Missing data	1.19 (0.69-2.05)	1.49 (0.55-4.03)	1.06 (0.51-2.16)	2.43 (0.74-8.01)	1.07 (0.52-2.20)	2.32 (0.71-7.63)
**Smoking habit**						
Never or almost never	Ref	Ref	Ref	Ref	Ref	Ref
Stopped smoking	0.85 (0.52-1.37)	2.91 (1.69-5.00)	0.85 (0.52-1.40)	3.09 (1.73-5.52)	0.85 (0.51-1.40)	3.05 (1.70-5.48)
Currently smoking	0.90 (0.62-1.33)	1.26 (0.64-2.45)	1.03 (0.69-1.52)	1.30 (0.66-2.58)	1.03 (0.69-1.52)	1.26 (0.64-2.51)
Missing data	1.28 (0.77-2.14)	0.71 (0.18-2.88)	1.08 (0.55-2.11)	0.35 (0.07-1.83)	1.08 (0.55-2.11)	0.35 (0.07-1.84)
**Physical activity**						
Less than once per week	1.76 (1.46-2.12)	1.82 (1.27-2.62)	1.32 (1.09-1.60)	1.36 (0.93-1.99)	1.31 (1.07-1.59)	1.30 (0.89-1.92)
Once or more per week	Ref	Ref	Ref	Ref	Ref	Ref
Missing data	1.26 (1.06-1.50)	1.15 (0.80-1.65)	1.03 (0.86-1.24)	0.97 (0.67-1.40)	1.05 (0.87-1.27)	0.88 (0.60-1.29)
**Frequency of social participation per year**						
1st quartile	1.54 (1.24-1.92)	2.17 (1.36-3.48)			1.03 (0.82-1.30)	1.48 (0.90-2.43)
2nd quartile	1.14 (0.89-1.47)	1.30 (0.75-2.26)			0.93 (0.72-1.20)	1.06 (0.61-1.86)
3rd quartile	1.17 (0.92-1.48)	1.44 (0.85-2.42)			1.05 (0.83-1.34)	1.32 (0.78-2.23)
4th quartile	Ref	Ref			Ref	Ref
Missing data	1.24 (1.00-1.54)	1.89 (1.19-3.01)			0.94 (0.75-1.18)	1.57 (0.97-2.54)

CI, confidence interval; GDS, Geriatric Depression Scale; PR, prevalence ratio.

In model 1, we controlled for risk factors of diseases, laughter, depression, age, body mass index, drinking habit, smoking habit, and physical activity.

In model 2, social participation was added to the variables.
