# Supplementary material for: Laughter is the Best Medicine? A Cross-Sectional Study of Cardiovascular Disease Among Older Japanese Adults
Source: J Epidemiol. 2016 Oct 5;26(10):546–52. doi: 10.2188/jea.JE20150196 (PMC5037252; doi:10.2188/jea.JE20150196)
Supplement: Abstract in Japanese. [file je-26-546-s006.pdf]

笑いは癒しになるか？　日本の高齢者の循環器疾患に関する横断研究

林慧[1]、カワチ・イチロー[2]、大平哲也[3]、近藤克則[4,5]、白井こころ[6]、近藤尚己[7]

[1]東京大学医学部医学科、[2]ハーバード大学公衆衛生大学院、[3]福島県立医科大学　医学部疫学講座、[4]日本福祉大学、[5]千葉大学 予防医学センター 社会予防医学研究部門、[6]琉球大学法文学部人間科学科、[7]東京大学大学院医学系研究科

背景：普段の笑いの頻度と心疾患および脳卒中有病の関連について、地域在住の日本の高齢者を対象として調査した。
方法：日本老年学的評価研究（JAGES）の 2013 年度の調査の対象者のうち 65 才以上の 20,934 人（男性 10,206 人、女性 10,728）のデータを分析した。郵送法の自記式調査により笑いの頻度、BMI、人口統計情報およびライフスタイル関連の要因、循環器疾患、高脂血症、高血圧、抑うつ症状についての情報を収集した。
結果：高脂血症、高血圧、抑うつ症状、BMI、その他の要因を調整した上でも、ほとんど笑わない群の心疾患の有病割合はほぼ毎日笑う群に比べて 1.21 倍　(95%信頼区間：1.03-1.41)　高かった。 同様に脳卒中の有病割合は 1.60 倍　(95% 信頼区間：1.24-2.06）高かった。
結論：普段よく笑う高齢者は循環器疾患（心疾患または脳卒中）が少ないという関連が見られた。この関連は抑うつ症状など要因では説明がつかなかった。

キーワード：笑い、高齢化、脳卒中、循環器疾患、日本
